# Quantitative Analysis of Foveal Microvascular Differences in Diabetic Macular Edema with and without Subfoveal Neuroretinal Detachment

**DOI:** 10.1155/2020/2582690

**Published:** 2020-02-26

**Authors:** Gao Jian, Xu Ya Jing, Li Yang, Liu Lun

**Affiliations:** Department of Ophthalmology, The First Affiliated Hospital of Anhui Medical University, Hefei, Anhui, China

## Abstract

**Purpose:**

This study is aimed at quantifying the difference of the foveal microvasculature in the eyes with diabetic macular edema (DME) with and without subfoveal neuroretinal detachment (SND+ and SND-, respectively).

**Methods:**

This retrospective, cross-sectional study included 48 eyes from 42 patients with DME (20 SND+ and 28 SND- eyes). Data collection included fundus color photographs, optical coherence tomography angiography (OCTA), and best-corrected visual acuity. The following parameters were evaluated with OCTA: foveal avascular zone (FAZ) parameters and vessel density in a width of 300 *μ*m around the FAZ, superficial capillary plexus, deep capillary plexus (DCP), and choriocapillary plexus. The number of retinal hyperreflective spots (HRS) and the area of SND in the central 3 mm were evaluated at 0 degrees using B-scans.

**Results:**

Parafoveal vessel densities of DCP were significantly lower in SND+ than in SND- eyes (*p* < 0.001). The number of HRS was significantly higher in SND+ than in SND- eyes (*p* < 0.001). The number of HRS was significantly higher in SND+ than in SND- eyes (*r* = 0.389, *p* < 0.001). The number of HRS was significantly higher in SND+ than in SND- eyes (

**Conclusion:**

DME with SND correlated with larger numbers of HRS and significant macular microvascular impairment in the DCP. The pathophysiology of decline of parafoveal vessel density in the DCP with an increase in the number of HRS in the eyes with DME with SND needs further investigation.

## 1. Introduction

Diabetic macular edema (DME) is primarily responsible for vision loss in patients with diabetic retinopathy (DR) and represents a significant public health issue [1, 2]. DME obtained by optical tomography (OCT) may vary and may be classified into three patterns: sponge-like swelling, cystoid macular edema, and subfoveal neuroretinal detachment (SND) [3–6]. Among these three patterns, studies on SND have captured the interest of clinicians. Some studies have shown a poorer visual prognosis, and response to antivascular endothelial growth factor (VEGF) therapy has been reported in the eyes with SRD [6, 7]. However, there is recent evidence that the presence of central subretinal fluid was associated with a larger anatomic response [8], and the data from the RIDE/RISE trial showed that the eyes with subretinal fluid were nearly 2.5 times more likely to achieve a central foveal thickness of 250 *μ*m or less at 2 years [9]. As a specific pattern of DME, different hypotheses of the formation of SND have been postulated. Leakage from the retinal or choroidal circulation into the subretinal space that exceeds the reabsorption capacity was considered to be the main mechanism [5, 10]. Moreover, a decrease of choroid blood flow, which may cause retinal pigment epithelium dysfunction, was found in patients with DME [9]. However, the authors did not compare the choroid blood flow between the eyes with DME with and without SND [11]. SND can disappear before or after the reabsorption of the leakage, suggesting that the formation of SND is not related to the severity or duration of DME [5]. Nevertheless, the relation between the pathogenesis and foveal microvasculature impairment of an SRD remains undetermined.

Chronic hyperglycemia leads to narrowing or occlusion of the retinal capillaries, tissue hypoxia, and increased VEGF levels [12]. Macular edema results from abnormal permeability [13]. Previous studies have reported on the changes in the foveal avascular zone (FAZ) shape and the impairment of foveal microcirculation, which showed macular ischemia in the eyes with DR [14–16]. However, little is known regarding the foveal microvascular impairment of DME as shown by different OCT types. The recent development of optical coherence tomography angiography (OCTA) has allowed the acquisition of good reproducibility and repeatability of the retinal microvascular images in a safe, rapid, and noninvasive manner [17]. Previous reports have demonstrated the ability of OCTA to visualize and quantify different layers of the retinal capillary network in patients with DR [14–16]. The main purpose of this study was to quantitatively analyze and compare the foveal microvascular differences between the eyes with DME with and without SND (SND+ and SND-, respectively) using OCTA.

## 2. Materials and Methods

### 2.1. Study Sample

This study was a cross-sectional, comparative, retrospective, and observational case evaluation of images and clinical charts of 48 eyes from 44 patients with DME who presented at the Ophthalmology Department of the First Affiliated Hospital of the Anhui Medical University. The study was performed in accordance with the tenets of the Declaration of Helsinki and was approved by the institutional review board of the First Affiliated Hospital of Anhui Medical University. All participants underwent ophthalmic examination, including measurement of best-corrected visual acuity (BCVA), fundus biomicroscopy examination, color fundus photography, measurement of intraocular pressure, and OCTA. Exclusion criteria were a history of uncontrolled glaucoma or ocular hypertension, a history of ocular surgery or any other retinal treatment (intravitreal injections, laser), iris neovascularization, and ischemic maculopathy.

### 2.2. Foveal Tomographic and Angiographic Imaging by OCTA

Foveal tomographic and angiographic images were obtained with the AngioVue OCTA device (Optovue, Inc., CA, USA). We obtained one linear scan at 0 degrees centered on the fovea in Enhanced Imaging mode and a 3 mm × 3 mm angiographic scan centered on the fovea. We excluded the eyes with poor image quality when the following symptoms were presented: media opacity obscuring the view of the microvasculature, presence of blink artifacts, and poor fixation leading to motion artifacts. Images of the superficial capillary plexus (SCP) and deep capillary plexus (DCP) and the choriocapillary network were generated automatically using the built-in software algorithm. The device automatically outlined the boundaries of the superficial retinal capillary network extended from 3 *μ*m below the internal limiting membrane to 15 *μ*m below the inner plexiform layer. The deep retinal capillary network extended from 15 *μ*m to 70 *μ*m below the inner plexiform layer. The choriocapillary network extended from 30 *μ*m to 60 *μ*m below the retinal pigment epithelium layer. Sometimes, an error in automatic segmentation occurred; in these cases, we manually corrected the entire scan volume.

### 2.3. FAZ and Foveal Microcirculation Parameters Collection

A series of parameters in the foveal area were obtained using the built-in AngioAnalytics software (version 2017.1.0.151; Optovue, Fremont, California, USA). In the FAZ quantitative analysis, the area, perimeter, and the acircularity index (AI) were evaluated as in a previous study [18]. The AI was measured using the following equation: AI = perimeter calculated/standard circular perimeter of the equal area. The foveal microcirculation parameters included the vessel density of the full retina in a width of 300 *μ*m around the FAZ (FD-300) and the parafoveal vessel density for the SCP and DCP. Vessel density of the choriocapillary plexus (CCP) was calculated as the percentage of the area occupied by blood vessels using the following formula: CCP = area occupied by vasculature flow/area of the central 3 mm around the fovea [19].

Central macular thickness (CMT) and intraretinal thickness (IRT) were automatically measured within the central 1 mm around the fovea including the SND. IRT was calculated from the inner limiting membrane to the outer plexiform layer. The following parameters were also measured: the number of the retinal hyperreflective spots (HRS) and the area of SND (both calculated in the area of 3 mm centered on the fovea) on B-scans passing through the center of the fovea at 0 degrees [20].

### 2.4. Statistical Analysis

For all statistical analyses, SPSS software for Windows, version 21.0 (IBM Corp., Armonk, NY, USA), was used. Normality of data was assessed using the Shapiro-Wilk test. All data are shown as the mean ± standard deviation (SD), median and interquartile range (IQR, 25th-75th percentile), or percentages where appropriate. The Student's *t*-test or the Wilcoxon rank test were used to assess the differences between the numerical data, depending on their distribution. Correlations between the DCP and the number of HRS or the SND area were examined by using the Spearman rank correlation analysis. All statistical tests were considered significant when the *p* value was <0.05.

## 3. Results

### 3.1. Demographics and Clinical Characteristics

A total of 48 eyes (28 and 20 eyes with and without SND) from 42 patients were evaluated, and the baseline clinical and demographics characteristics of these patients are shown in Table 1. There were no significant differences in age, disease duration, BMI, Hba1c, CKD stage, BCVA, and SQI between patients with SND+ and SND- (Table 1).

### 3.2. Comparisons of the OCTA Measurements between the Eyes with and without SND

**Figure 1:**
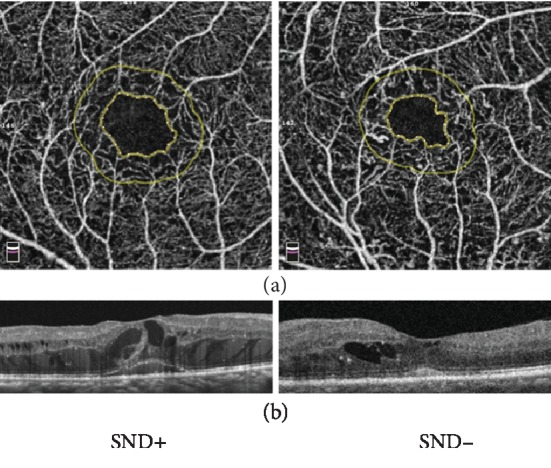
(a) Representative samples of the foveal avascular zone (FAZ) measurements including the area, perimeter, AI, and FD-300 at the foveal avascular zone for the eyes with and without subfoveal neuroretinal detachment (SND). (b) B-scans showing diabetic macular edema with or without SND.

**Figure 2:**
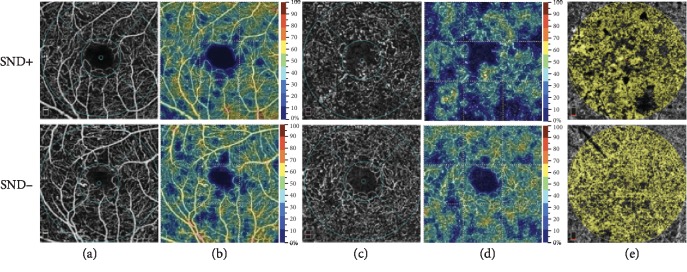
Representative samples of the foveal microvascular parameters for the eyes with and without subfoveal neuroretinal detachment (SND): (a) shows the parafoveal vessel density in the superficial capillary plexus (SCP); (b) shows the vessel density in the SCP using color maps; (c) shows the parafoveal vessel density in the deep capillary plexus (DCP); (d) shows the vessel density in the DCP using color maps; (e) shows vessel density in the choriocapillary plexus.

Mean values or median and interquartile range of FAZ area, FAZ perimeter, AI, vessel density of FD-300, parafoveal vessel density of SCP and DCP, and vessel density of CCP are listed in Table 2. The parafoveal vessel density of DCP was significantly higher in the SND- eyes than in the SND+ eyes (*p* < 0.001) (Table 2). No significant differences in the FAZ area, FAZ perimeter, AI, vessel density of FD-300 and CCP, and parafoveal vessel density of SCP were observed between the SND- and SND+ eyes (all *p* > 0.05) (Table 2). The top row in Figure 1 presents a representative sample of FAZ measurements in the eyes with and without SND.  Figure 2 presents a representative sample of parafoveal vessel density in the SCP (the first column), DCP (the third column), and CCP (the fifth column) in the eyes with and without SND. Color maps were used to show the difference in vessel density in the SCP (the second column) and DCP (the fourth column).

**Figure 3:**
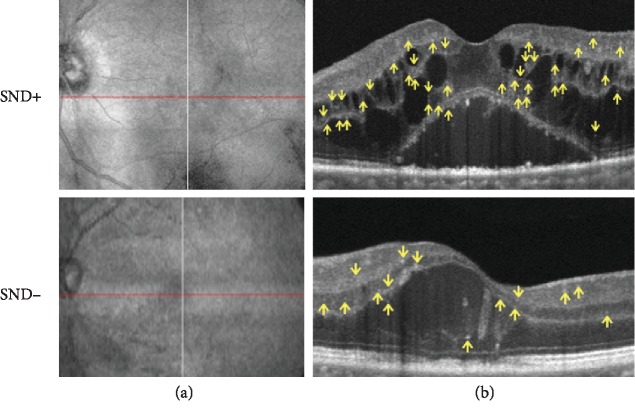
Infrared reflectance image from the optical coherence tomography angiography image; the red line indicates the position of the B-scan (a). B-scan obtained in the enhanced imaging mode showing cystoid macular edema, subfoveal neuroretinal detachment, and the number of hyperreflective retinal spots (HRS) as indicated by the yellow arrows within the central 3 mm (b).

Table 3 shows the following parameters: CMT, IRT, SND area, and number of HRS at 0 degrees. CMT and IRT were not significantly different between the SND+ and SND- eyes (*p* = 0.109) (Table 3). The number of HRS at 0 degrees was significantly higher in the SND+ than in the SND- eyes (*p* < 0.001) (Table 3) (Figure 3).

### 3.3. Correlations between the Parafoveal Vessel Density in DCP, the Numbers of HRS, and the Area of SND

**Figure 4:**
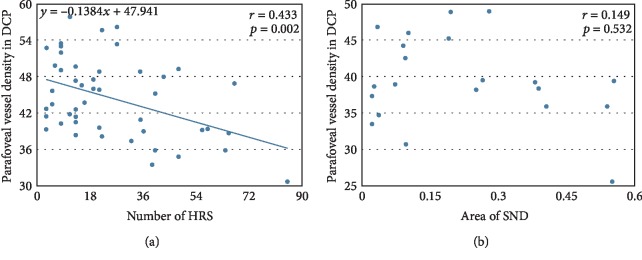
(a) Scatterplots illustrating the linear associations between the parafoveal vessel density in the deep capillary plexus (DCP) and the numbers of hyperreflective retinal spots (HRS) in all eyes. (b) Scatterplots illustrating the linear associations between the parafoveal vessel density in DCP and the area of SND in SND+ eyes. A value of *p* < 0.05 was considered statistically significant. *r*, correlation coefficient from the Spearman rank correlation analysis.

**Figure 5:**
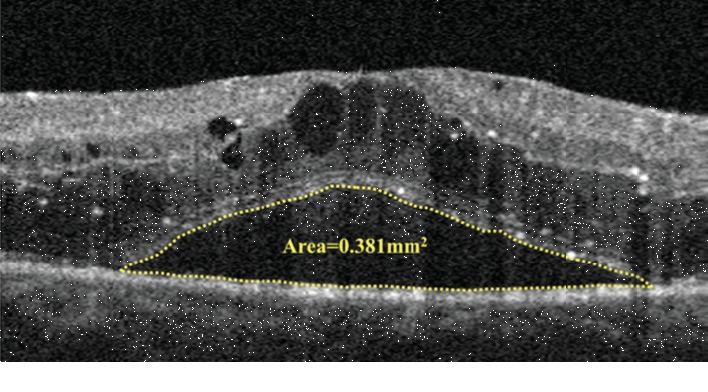
The area of subfoveal neuroretinal detachment was obtained by using the built-in AngioAnalytics software (calculated in the area of 3 mm centered on the fovea) on B-scan passing through the center of the fovea at 0 degrees.

Figure 4(a) shows a statistically significant negative correlation between the parafoveal vessel density in DCP and the numbers of HRS in all eyes (Spearman's correlation, *r* = 0.433, *p* = 0.002). However, no significant correlation was observed between the parafoveal vessel density in DCP and the SND area (Figure 5) in the SND+ eyes (Spearman's correlation, *r* = 0.149, *p* = 0.532) (Figure 4(b)).

## 4. Discussion

Our aim was to obtain more details to better understand the clinical characteristics of the eyes with SND. Therefore, we evaluated and compared the morphologic and microvascular characteristics of the eyes with center-involving DME with and without SND. The average CMT was higher in the SND+ than in the SND- eyes, whereas there was no significant difference between the two groups. There was also no significant difference in intraretinal thickness in the perifoveal region, which was similar with the findings of previous studies [21].

Previous studies have reported poorer vision gains in the eyes with SND than in the eyes with other types of DME with treatment using anti-VEGF drugs [5, 6, 22, 23]. Seo et al. found that disruption of the photoreceptor integrity at the baseline occurred more frequently in the eyes with SND+, which was correlated with poorer visual outcome [6]. Nevertheless, the pathophysiology of the impairment of photoreceptors with SND in DME is not yet fully understood. Usui et al. found that DCP is critical to meet the high energy demands of the highly specialized photoreceptor synapses in the outer platform layer [24]. Scarinci et al. also highlighted the contribution of DCP to the energy requirements of the photoreceptors and the outer retina in diabetic macular ischemia [25]. Interestingly, we calculated the vessel density in SCP, DCP, CCP, and FD-300 using OCTA. Nevertheless, only the parafoveal vessel density in DCP showed a significant decrease in the SND+ than in the SND- eyes. We are extrapolating from these findings to propose that the photoreceptor compromise and poor visual outcome in the eyes with SND+ caused by DCP ischemia. This decreased flow in the DCP might be an important direction for future studies exploring the pathophysiology of SND in the eyes with DME. However, recent evidence from https://DRCR.net showed that glycemic control was associated with the magnitude of vision improvement following anti-VEGF therapy in 2 years [8]. Thus, further prospective and longitudinal further analysis is necessary.

HRS on B-scan have been suggested as an imaging marker of retinal inflammation which represented activated microglial cells in the retina [20, 26–28]. A series of characteristics have been previously examined, including a size of ≤30 *μ*m, absence of back shadowing, and reflectivity similar to that of the nerve fiber layer [28]. Our results showed a significant increase in the number of HRS in the SND+ than in the SND- eyes, which was in line with previous findings [20, 21]. The presence of an SND was significantly correlated with a higher intravitreal level of interleukin-6, which was indicated as a heightened “inflammatory condition” in a previous study [29]. Moreover, in our work, there was a negative correlation between the number of HRS and the parafoveal vessel density in DCP. However, it was not clear whether the high inflammatory state led to macular capillary closure and decreased perfusion in the DCP or whether it was DCP ischemia in the eyes with SND that caused cell damage and attracted scavenging cells to the retina, which finally became a source of inflammation. This hypothesis must be further explored in future studies.

The choroidal circulation in the foveal region in patients with DME is impaired, but the difference in choroidal circulation between the eyes with and without SND has not been reported [9]. Our data confirmed that there was no significant difference in the vessel density of the choroidal circulation in the foveal area.

Gaucher et al. found that SND can appear very early in the evolution of DME, and SND may be absorbed despite DME worsening [5]. These findings suggested that SND was not associated with severe of DME. In the present study, our data showed that there was no correlation between the parafoveal vessel density in the DCP and the area of SND. Therefore, the amount of leakage in the subretinal space in the eyes with SND+ does not seem to be attributed to DCP ischemia only, which needs further study.

Our work had some limitations as it was a retrospective study and the present Angio Vue system examined the data from two retinal plexuses (only SCP and DCP). A longitudinal and prospective study evaluating these specific characteristics of the foveal microcircular parameters may provide more information on the pathophysiology and the therapeutic outcomes in DME with SND.

## 5. Conclusion

In conclusion, this study suggested that DME in the eyes with SND+ showed a more severe ischemic state in the DCP of the macular area and resulted in a high number of HRS. Moreover, the significant decline of parafoveal vessel density in the DCP with increasing number of HRS in the eyes with DME with SND suggested that specific flow alterations at this retinal capillary layer may have functional consequences for photoreceptors and warrant further investigation.

## Figures and Tables

**Table 1:** Demographics and clinical characteristics of the study participants.

Variable	Subfoveal neuroretinal detachment		*p* value
	No	Yes	
Patients, *n*	25	19	
Eyes, *n*	28	20	
Sex			
Female, *n*	9	9	
Male, *n*	16	10	
Age (y), mean ± SD	49.24 ± 10.89	52.63 ± 7.81	0.257
Disease duration (y), mean ± SD	8 (5-10)	9 (5-10)	0.933
BMI, mean ± SD	23.64 ± 2.41	24.32 ± 3.01	0.612
Hba1c, mean ± SD	9.52 ± 1.75	9.62 ± 1.49	0.769
Insulin, *n* (%)	25 (100%)	19 (100%)	
CKD stage, *n* (%)			0.902
Stage 1	9 (36%)	7 (37%)	
Stage 2	10 (40%)	5 (26%)	
Stage 3	1 (4%)	2 (11%)	
Stage 4	1 (4%)	1 (5%)	
Stage 5	0	0	
Missing	4 (16%)	4 (21%)	
Hemodialysis	0	0	
BCVA (logMAR), mean ± SD	0.8 (0.6-0.9)	0.8 (0.7-0.875)	0.617
SQI, mean ± SD	6.5 (5.25-7.00)	7.00 (6.00-7.75)	0.182

BMI: body mass index; CKD: chronic kidney disease.

**Table 2:** Comparisons of parameters at the foveal avascular zone and the foveal microcirculation between the eyes with and without subfoveal neuroretinal detachment.

Variable	Subfoveal neuroretinal detachment		*p* value
	No	Yes	
FAZ area (mm^2^), mean ± SD	0.26 ± 0.10	0.33 ± 0.16	0.082
FAZ perimeter (mm), mean ± SD	2.12 ± 0.40	2.58 ± 1.28	0.078
AI, mean ± SD	1.15 (1.10-1.20)	1.19 (1.13-1.27)	0.201
Parafoveal vessel density			
SCP (%), mean ± SD	37.86 ± 4.29	38.27 ± 4.24	0.750
DCP (%), mean ± SD	47.47 ± 5.43	40.19 ± 5.15	<0.001
Vessel density			
FD-300 (%), mean ± SD	43.10 ± 5.98	43.41 ± 3.33	0.836
CCP (%), mean ± SD	56.60 ± 4.19	54.96 ± 6.34	0.286

**Table 3:** Comparisons of the morphologic measurements between the eyes with and without subfoveal neuroretinal detachment.

Variable	Subfoveal neuroretinal detachment		*p* value
	No	Yes	
CMT (*μ*m), mean ± SD	378.68 ± 110.27	441.00 ± 157.98	0.109
IRT (*μ*m), mean ± SD	67.25 ± 21.75	66.85 ± 19.24	0.948
Area of SND (mm^2^), mean ± SD		0.147 (0.046-0.356)	
HRS, mean ± SD	13.00 (6.50-20.75)	37.50 (21.25-57.50)	<0.001

## Data Availability

Researchers can access the data supporting the conclusions of this study directly via the authors. Liu Lun and Jian Gao can be contacted to request the data. Email: liulundoc@126.com or shuijinglovegj@126.com.

## References

[1] Tranos P. G., Wickremasinghe S. S., Stangos N. T., Topouzis F., Tsinopoulos I., Pavesio C. E. (2004). Macular edema. *Survey of Ophthalmology*.

[2] Antonetti D. A., Klein R., Gardner T. W. (2012). Diabetic retinopathy. *New England Journal of Medicine*.

[3] Catier A., Tadayoni R., Paques M. (2005). Characterization of macular edema from various etiologies by optical coherence tomography. *American Journal of Ophthalmology*.

[4] Scholl S., Augustin A., Loewenstein A., Rizzo S., Kuppermann B. D. (2011). General pathophysiology of macular edema. *European Journal of Ophthalmology*.

[5] Gaucher D., Sebah C., Erginay A. (2008). Optical coherence tomography features during the evolution of serous retinal detachment in patients with diabetic macular edema. *American Journal of Ophthalmology*.

[6] Seo K. H., Yu S. Y., Kim M., Kwak H. W. (2016). Visual and morphologic outcomes of intravitreal ranibizumab for diabetic macular edema based on optical coherence tomography patterns. *Retina*.

[7] Shimura M., Yasuda K., Yasuda M., Nakazawa T. (2013). Visual outcome after intravitreal bevacizumab depends on the optical coherence tomographic patterns of patients WITH diffuse diabetic macular edema. *Retina*.

[8] Bressler S. B., Odia I., Maguire M. G. (2019). Factors associated with visual acuity and central subfield thickness changes when treating diabetic macular edema with anti–vascular endothelial growth factor therapy: an exploratory analysis of the protocol T randomized clinical trial. *JAMA Ophthalmology*.

[9] Sophie R., Lu N., Campochiaro P. A. (2015). Predictors of functional and anatomic outcomes in patients with diabetic macular edema treated with ranibizumab. *Ophthalmology*.

[10] Marmor M. F., Marmor M. F., Wolfensberger T. J. (1998). Control of subretinal fluid and mechanisms of serous detachment. *The retinal pigment epithelium: function and disease*.

[11] Nagaoka T., Kitaya N., Sugawara R. (2004). Alteration of choroidal circulation in the foveal region in patients with type 2 diabetes. *British Journal of Ophthalmology*.

[12] Frank R. N. (2007). Diabetic retinopathy. *New England Journal of Medicine*.

[13] Ishida S., Usui T., Yamashiro K. (2003). VEGF164 is proinflammatory in the diabetic retina. *Investigative Ophthalmology & Visual Science*.

[14] Freiberg F. J., Pfau M., Wons J., Wirth M. A., Becker M. D., Michels S. (2016). Optical coherence tomography angiography of the foveal avascular zone in diabetic retinopathy. *Graefe's Archive for Clinical and Experimental Ophthalmology*.

[15] Bradley P. D., Sim D. A., Keane P. A. (2016). The evaluation of diabetic macular ischemia using optical coherence tomography angiography. *Investigative Ophthalmology & Visual Science*.

[16] Hwang T. S., Gao S. S., Liu L. (2016). Automated quantification of capillary nonperfusion using optical coherence tomography angiography in diabetic retinopathy. *JAMA Ophthalmology*.

[17] Spaide R. F., Fujimoto J. G., Waheed N. K. (2015). Optical coherence tomography angiography. *Retina*.

[18] Liu L., Jian Gao, Bao W. (2018). Analysis of foveal microvascular abnormalities in diabetic retinopathy using optical coherence tomography angiography with projection artifact removal. *Journal of Ophthalmology*.

[19] Al-Sheikh M., Phasukkijwatana N., Dolz-Marco R. (2017). Quantitative OCT angiography of the retinal microvasculature and the choriocapillaris in myopic eyes. *Investigative Ophthalmology & Visual Science*.

[20] Vujosevic S., Torresin T., Bini S. (2017). Imaging retinal inflammatory biomarkers after intravitreal steroid and anti-VEGF treatment in diabetic macular oedema. *Acta Ophthalmologica*.

[21] Vujosevic S., Torresin T., Berton M., Bini S., Convento E., Midena E. (2017). Diabetic macular edema with and without subfoveal neuroretinal detachment: two different morphologic and functional entities. *American Journal of Ophthalmology*.

[22] Kim M., Lee P., Kim Y., Yu S. Y., Kwak H. W. (2011). Effect of intravitreal bevacizumab based on optical coherence tomography patterns of diabetic macular edema. *Ophthalmologica*.

[23] Ashraf M. (2017). Functional and anatomic outcomes in patients with serous retinal detachment in diabetic macular edema treated with ranibizumab. *Investigative Opthalmology & Visual Science*.

[24] Usui Y., Westenskow P. D., Kurihara T. (2015). Neurovascular crosstalk between interneurons and capillaries is required for vision. *Journal of Clinical Investigation*.

[25] Scarinci F., Nesper P. L., Fawzi A. A. (2016). Deep retinal capillary nonperfusion is associated with photoreceptor disruption in diabetic macular ischemia. *American Journal of Ophthalmology*.

[26] Vujosevic S., Bini S., Midena G., Berton M., Pilotto E., Midena E. (2013). Hyperreflective intraretinal spots in diabetics without and with nonproliferative diabetic retinopathy: an in vivo study using spectral domain OCT. *Journal of Diabetes Research*.

[27] Vujosevic S., Bini S., Torresin T. (2017). Hyperreflective retinal spots in normal and diabetic eyes: B-scan and en face spectral domain optical coherence tomography evaluation. *Retina*.

[28] Vujosevic S., Berton M., Bini S., Casciano M., Cavarzeran F., Midena E. (2016). Hyperreflective retinal spots and visual function after anti-vascular endothelial growth factor treatment in center-involving diabetic macular edema. *Retina*.

[29] Sonoda S., Sakamoto T., Yamashita T., Shirasawa M., Otsuka H., Sonoda Y. (2014). Retinal morphologic changes and CONCENTRATIONS of cytokines in eyes with diabetic macular edema. *Retina*.

